# Strategies for Reuse of Skins Separated From Grape Pomace as Ingredient of Functional Beverages

**DOI:** 10.3389/fbioe.2020.00645

**Published:** 2020-06-26

**Authors:** Carmela Gerardi, Leone D'amico, Danilo Migoni, Angelo Santino, Antonio Salomone, Maria A. Carluccio, Giovanna Giovinazzo

**Affiliations:** ^1^CNR-ISPA, Institute of Sciences of Food Production, National Research Council, Lecce, Italy; ^2^Department of Biological and Environmental Sciences and Technologies (DiSTeBA), University of Salento, Lecce, Italy; ^3^CNR-IFC, Institute of Clinical Physiology, National Research Council, Lecce, Italy

**Keywords:** polyphenols, pomace, wine by-product, waste, functional beverages

## Abstract

Wine grape pomace, the by-product of wine making, is a source of polyphenols, metals, and organic acids, and may be exploited for the production of functional beverages. Among red wines, Primitivo and Negramaro varieties possess an interesting amount of polyphenolic compounds and other chemicals. Consequently, study of the biological activity of Primitivo and Negramaro vinification by-products is of great interest as well as optimizing the extraction of its bioactive components. In order to stabilize the grape pomace, different methods of drying grape pomace were tested. After stabilization of the pomace, the grape skins were manually separated from the seeds and any woody parts. The chemical characterizations of acidified alcoholic (methanol/ethanol) and water extracts and either microwave-assisted or ultrasound-assisted extractions of separated grape skins were compared. Besides that, the *in vitro* antioxidant activity of wine pomace skin extracts was also investigated as Trolox equivalents antioxidant capacity (TEAC) and oxygen radical absorbance capacity (ORAC). Overall, the alcoholic extractions were found to be the most effective for recovering phenolic compounds, when compared with those in water. Ultrasound- and microwave-assisted extraction of pomace skin using acidified water allowed the highest TEAC value. Taking into account the water extraction result, in order to reuse grape pomace skins to produce a functional beverage, we utilized them in combination with black tea, karkadè (*Hibiscus sabdariffa* L.), or rooibos (*Aspalathus linearis* Burm.) to produce an infusion. The combination of grape skins and black tea showed the highest ratio of total phenol content to antioxidant activity. Moreover, skin isolated from pomace, with or without black tea infusions, were shown to have anti-inflammatory capacity in human cell culture. Our results raise the value of grape skin pomace as a rich source of bioactive compounds with antioxidant and anti-inflammatory activity and suggest its exploitation as an ingredient for functional beverages.

## Introduction

Grape pomace is the by-product of the winemaking process, representing 20% of the processed grape weight (Beres et al., 2017). Grape pomace can be exploited as a source of various ingredients for the human diet, such as antioxidants, dietary fibers, and phenolic extracts, to produce novel foods. The by-product can thus re-enter the food cycle, avoiding environmental complications, due to the phenols (Tang et al., 2018). There is growing interest in the potential food applications of phenolic compounds therefore, the reuse of by-products could provide a new food chain. The vast amounts of winemaking by-products and the seasonality of their production force the development of fast and economical methods that allow their stabilization and storage for later use. Winemaking by-products must be dried for further utilization. Drying methods have a great impact on the stability of bioactive compounds, directly influencing changes in the physicochemical properties of food ingredients.

The most utilized drying methods include, for example, hot-air/oven drying, low temperature-air drying, and freeze drying (Barba et al., 2016; Medina-Torres et al., 2017). Except for freeze drying, these conventional drying methods are easier and have lower production costs. The optimal drying conditions for the storage of grape pomace for further application need further investigation. Moreover, optimization of the experimental conditions for extraction of phenolics from grape pomace is a key issue for future industrial developments.

Industrial extraction from the grape pomace is a process that combines water with other organic solvents (Chemat et al., 2017). In order to meet the ongoing demands for minimally processed by-products and meet the requirements of a green extraction concept, alternative extraction methods have been pointed out. Microwave- and ultrasound-assisted extraction have been described as good alternatives to conventional techniques due to some advantages such as shorter extraction time, lower solvent requirement, and a higher extraction rate (Chemat et al., 2017). UAE treatment offers the advantage of greater permeation of the solvent into dried tissues, shorter incubation, higher yields and reproducibility, high processing throughput, extraction of heat labile components, and less energy needed for extraction (Roselló-Soto et al., 2015). The MAE extraction process is another green method based on the polarity of the compounds. Due to the faster heating for the extraction of bioactive plant substances, the reduction of thermal gradients and the increase of the yield of the extracts, the MAE process is widely studied for the extraction of bioactive molecules.

Grape by-products contain a variety of phytochemicals, especially phenols and flavonoids like anthocyanins, stilbenes, flavonols, and flavanols, and essential minerals (Cádiz-Gurrea et al., 2017; Tang et al., 2018). These bioactive molecules possess different biological activities such as antibacterial, antitumor, antioxidant, anti-inflammatory effects (Tomé-Carneiro et al., 2012; Xu et al., 2014; Vaisman and Niv, 2015; Ismail et al., 2016; Ferri et al., 2017; Iannone et al., 2017; Leone et al., 2019) which are effective in preventing chronic diseases (Li et al., 2017; Tang et al., 2017; Zhao et al., 2017; Meng et al., 2018).

The most sustainable approach to exploitation of pomace could be its use as a raw material to produce ingredients for functional foods, and pharmaceuticals (Meral and Doğan, 2013; Wittenauer et al., 2016). Our previous studies on grape skin and data from the literature show that the composition and amounts of polyphenols in grape berries can be influenced by different factors, among which genotype usually has the greatest impact (Giovinazzo and Grieco, 2015; Calabriso et al., 2016b). Moreover, viticulture and vinification practices also affect the polyphenol extractability in wine and grape pomace (Grieco et al., 2019). Previous studies have shown that red grape skin polyphenols counteract the atherosclerotic process through reduced expression of endothelial adhesion molecules, chemokines, and matrix metalloproteinases (Calabriso et al., 2016a,b). This study aimed to: (i) investigate the influence of various drying conditions on the antioxidant activity and polyphenol stability of grape pomace skin for potential application in functional foods and beverages; (ii) compare the antioxidant capacity of pomace skin from different varieties, their phenolic and flavonoid quantification and characterization after organic solvent and water extractions; (iii) evaluate the phenolic and flavonoid content and antioxidant activity of grape pomace skin infusion alone or combined with black tea, karkadè (*Hibiscus sabdariffa* L.), or rooibos (*Aspalathus linearis* Burm.); (iv) assess the anti-inflammatory capacity in human cell culture of grape pomace skin infusion alone or combined with black tea. Our findings aimed to raise the value of grape skin pomace as a rich source of bioactive compounds with health properties and suggest its exploitation as an ingredient for functional beverages.

## Materials and Methods

### Reagents and Standards

Reagents were acquired from various suppliers: authentic standards of kuromanin (cyanidin 3-O-glucoside chloride), rutin (quercetin 3-O-rutinoside), chlorogenic acid (5-caffeoylquinic acid) from Extrasynthèse (Genay, France); gallic acid, Folin–Ciocalteu phenol reagent, Trolox [(S)-(-)-6-hydroxy-2,5,7,8 tetramethylchroman-2-carboxylic acid], fluorescein disodium, ABTS [2,2′-azino-bis (3-ethylbenzothiazoline-6-sulfonic acid)], AAPH [2,2′-azobis (2-methyl-propionamide)], acetonitrile, formic acid, ethanol, and organic acids (all HPLC grade) from Sigma-Aldrich (St. Louis, MO, USA). ICP-AES standards were obtained from Ultra Scientific Analytical Solutions (Bologna, Italy). Milli-Q water was used (Merck Millipore, Darmstadt, Germany).

### Raw Material and Sample Preparation

Four batches of wine pomace, *Vitis vinifera* varieties Primitivo (P), Negramaro (N), Negramaro/Lambrusco (N/L) (achieved after fermentation for red wine making), and Verdeca (B) (without fermentation, as it is used in white wine making) were obtained from a commercial winemaking facility located in Salento (*Cantine Due Palme*; southern area of the Apulia Region, Italy). The pomace was dried (a) in an oven at 50°C, (b) at room temperature, or (c) by a Freezone^®^ 2.5 model 76530 lyophilizer (Labconco Corp., Kansas City, MO, USA) until constant weight. Subsequently, the skins were manually recovered from the pomace samples and stored at −20°C until further processing. A sample of wet skins was stored at −20°C and utilized for comparison experiments. Commercial black tea leaves, dried hibiscus petals (*Hibiscus sabdariffa* L.), and rooibos tea (*Aspalathus linearis* Burm.) were purchased from a local supermarket. Three blends were prepared by mixing 50% dried skin from grape pomace with 50% black tea, hibiscus petals, or rooibos tea.

## Extraction Methods

### Extraction of Polyphenol Compounds in Organic Solvent

Polyphenol compounds were extracted from wet grape skins and skins separated from dried grape pomace by freezing the samples in liquid nitrogen and grinding with a blender until a fine powder was obtained. The samples (1 g) were treated with 10 mL of methanol:ethanol (80:20, v:v) and extracted at room temperature for 16 h in the dark under continuous stirring. Extraction mixtures were centrifuged (4,000 × g) for 5 min and the supernatants stored at −20°C until analysis.

### Extraction of Polyphenol Compounds in Acidified Water

Skins isolated from grape pomace and homogenized as described above (5 g and 10 g) were extracted in distilled water (100 mL) acidified with citric acid (0.001 M, final concentration) (acidified water) at room temperature for 16 h in the dark under continuous stirring. After centrifugation (4,000 × g for 5 min) of the extraction slurry, the supernatants were stored at −20°C until analysis.

### Ultrasound-Assisted Extraction

Ultrasound-assisted extraction (UAE) was carried out in an ultrasound bath (Labsonic Falc, LBS1-H3) at 35 kHz frequency and 88 W, at room temperature for 15 min. Samples of grape skin from dried pomace (5 g) were extracted with 100 mL of distilled water acidified as described above. Samples were centrifuged at 4000 × g for 5 min after ultrasound treatment or were left at room temperature for 16 h in the dark under continuous stirring and then centrifuged to remove cell debris as described above.

### Microwave-Assisted Extraction

Microwave-assisted extraction (MAE) was carried out in a Multiwave 3000 SOLV Microwave Reaction System (Anton Paar, Austria). Samples of dried skin pomace (5 g) were extracted by adding 100 mL of acidified water at room temperature and using fluctuating radiation to keep the temperature steady at a set value of 50°C and 200 W, as described by Drosou et al. (2015). After microwave treatment, the samples were centrifuged at 4,000 × g for 5 min or were left at room temperature for 16 h in the dark under continuous stirring and then centrifuged to recover supernatants.

### Infusion

Primitivo grape skins isolated from pomace as described above (P), black tea (T), karkadè (K), and rooibos (R) infusions were formulated as follows: 2 g of P; 1 g of P and 1 g of T (P/T); 1 g of P and 1 g of K (P/K); 1 g of P and 1 g of R (P/R). The formulations (2 g total) were infused in 100 mL of drinking water at a temperature of 90°C for 5 min; the infusions were acidified using 1 mL of lemon juice. The infusions were stored at −20°C until characterization analyses. The infusions were freeze-dried by a Freezone® 2.5 model 76530 lyophilizer (Labconco Corp., Kansas City, MO, USA) and stored at −20°C until biological activity analysis.

## Characterization of Alcoholic and Aqueous Extracts

### High Performance Liquid Chromatography (HPLC) Characterization of Anthocyanins

To quantify the anthocyanin molecules in both alcoholic and aqueous extracts from skins isolated from grape pomace, we performed HPLC analysis using an Agilent-1100 liquid chromatograph equipped with a DAD detector as described by Gerardi et al. (2015). Chromatograms were acquired at 520 nm. Quantification of total anthocyanins was performed by HPLC/DAD using a five-point regression curve (*r*^2^ ≥ 0.99) generated through the use of malvidin 3-*O*-glucoside (oenin) as a reference compound and expressed as oenin equivalents (OEs).

### HPLC Characterization of Phenolic Acids, Stilbenes, Flavanols, and Flavonols

Different phenolic compounds present in alcoholic and aqueous extracts were separated by RP-HPLC DAD (Agilent 1100 HPLC system, Santa Clara, CA, USA). The separation was performed as described by Calabriso et al. (2016b). The chromatographic analysis was based on the comparison of peak retention time with the retention time of external standards.

### HPLC Characterization of Organic Acids and Alcohols

Organic acids and alcohol were identified and quantified by the HPLC system (Agilent 1100 series) equipped with a refractive index detector (RID) for alcohol analysis, and a UV/Vis detector for analyzing organic acids at 210 nm onto an Aminex HPX-87H column (300 × 7.8 mm, 9 μm) (Bio-Rad, Hercules, CA, USA), at 55°C. The analytical method was the same as that reported by Gerardi et al. (2019).

### Metal Characterization

Element concentrations in grape pomace samples were measured using inductively coupled plasma atomic emission spectroscopy (ICP-AES). The spectrometer was an ICAP 6300 with Dual view, empowered by iTEVA software (Thermo Scientific, Waltham, MA, USA).

One gram of dried grape skin pomace or lyophilized infusion was treated and analyzed as described by Bruno et al. (2018), whereas the liquid samples were analyzed as they were. A blank sample was also prepared with the same solvents and preparation procedures used for the pomace samples: 4 mL of H_2_O_2_ and 6 mL of 69% HNO_3_, treatment at 180°C for 10 min in a START D microwave digestion system (Milestone, Italy), and dilution with superpure water to a final volume of 25 mL.

The spectrometer was previously calibrated for quantitative analysis with five standard solutions containing known concentrations (0.001, 0.01, 0.1, 0.5, and 1.0 mg/L) of the elements. The calibration lines showed correlation coefficients (r) >0.99 for all the measured elements. The results were expressed as the average of three different measurements, and the element concentrations were expressed as ppm (mg/kg of sample weight) for solid samples, and as mg/L for liquid samples. Std for blank sample: 0.003 (0 ± 0.003).

## Antioxidant Activity of Alcoholic and Aqueous Extracts

### Trolox Equivalent Antioxidant Capacity (TEAC) Assay

The TEAC assay was performed by the method described by Re et al. (1999), modified as reported by Scarano et al. (2018). Briefly, the ABTS radical cation was diluted in PBS (pH 7.4) to an absorbance of 0.40 at 734 nm. After the addition of 200 μL of diluted ABTS to 10 μL of Trolox standard or extract, the absorbance reading at 734 nm was taken 6 min after initial mixing using an Infinite 200 Pro plate reader (Tecan, Männedorf, Switzerland). The percentage inhibition of absorbance at 734 nm was calculated and plotted as a function of the concentration of Trolox, and the TEAC value expressed as Trolox equivalents (μmol) using Magellan v7.2 software.

### Oxygen Radical Absorbance Capacity (ORAC) Assay

The ORAC procedure was carried out following the procedure established by Dávalos et al. (2004). The assay was performed in 75 mM phosphate buffer (pH 7.4) at 37°C using 96-well plates and an Infinite 200 Pro plate reader (Tecan, Männedorf, Switzerland). The antioxidant capacity of 20 μL of extract from dried grape skin isolated from pomace was assayed by recording for 80 min the decay curves of fluorescein (70 nM final concentration) after the addition of a generator of radical species (AAPH, 12 mM final concentration). The antioxidant Trolox was used to make a standard curve (1–6 μM) and final ORAC values were expressed as μmol Trolox equivalents (TE)/g of dried weight of grape skins or μmol TE/L of aqueous extractions.

### Folin-Ciocalteu Assay

A rapid method (Magalhães et al., 2010) was used to assess the total phenols in alcoholic and water extracts from dried skins isolated from pomace in 96-well plates (Corning) using a microplate reader (Tecan, Infinite M200). Folin-Ciocalteu reagent (1:5, v/v) (50 μL) was placed in each well, and then 100 μL of sodium hydroxide solution (0.35 M) was added. The absorbance at 760 nm of the blue complex formed was monitored after 5 min. Gallic acid was used to obtain a calibration curve in the range from 2.5 to 40.0 mg/L (*R* ≥ 0.9997). The total phenol content of the samples was expressed as gallic acid equivalents.

## Biological Activity of Aqueous Extracts

### Cell Culture and Treatment

Human microvascular endothelial cell line (HMEC-1) was obtained from Dr. Thomas J. Lawley and cultured as described by Ades et al. (1992). Briefly, microvascular endothelial cells were cultured in MCDB-131 medium supplemented with 10% fetal calf serum (FCS), 2 mM glutamine, 1 μg/mL hydrocortisone (Sigma), 10 ng/mL human epidermal growth factor (Sigma), 50 U/mL penicillin, and 50 μg/mL streptomycin (Gibco BRL, Life Technologies, Paisley, UK). For treatments, confluent endothelial cells were shifted to MCDB-131 medium supplemented with 3% fetal bovine serum for 3 h and then treated for 2 h with aqueous extracts of grape skins infusion of Primitivo pomace (P) at several concentrations (1–5 mg/mL). Then, endothelial monolayers were stimulated with the cytokine tumor necrosis factor-α (TNF-α) (10 ng/mL) for an additional 16 h, after which cellular toxicity, inflammatory markers, and adhesion assays were evaluated.

### Monocytoid Cell Adhesion Assays

Monocytoid U937 cells were obtained through the American Tissue Culture Collection (Rockville, MD) and grown as described by Carluccio et al. (2003). Adhesion assays were performed as follows: HMEC-1 were grown to confluence in six-well tissue culture plates, after which TNF-α was added (16 h) to induce the expression of VCAM-1, in the presence or absence of aqueous extracts of grape skin infusion of Primitivo pomace (P) at several concentrations (1–5 mg/mL). Adhesion assays were performed as described by Carluccio et al. (2003). After 10 min, non-adhering cells were removed by gentle washing with cell culture medium, and the monolayers were fixed with 1% paraformaldehyde. The number of adherent cells was obtained by counting six different fields by using an ocular grid and a 20 × objective (0.16 mm^2^/field).

### Detection of Endothelial Cell Surface Molecules

HMEC-1 were grown to confluence in 96-well tissue culture plates. Then, the cultures were incubated in the presence or absence of aqueous extracts of grape skin infusion of Primitivo pomace (P), tea (T), or P/T (1:1) for 2 h and then stimulated with TNF-α for 16 h.

Assays of cell surface molecules were performed as previously described (Carluccio et al., 2003), by cell-surface enzyme immunoassays (EIA) with primary mouse anti-human monoclonal antibodies against VCAM-1 (Millipore) or ICAM-1 (HU5/3).

### Statistical Analysis

The experiments with skin pomace extracts were conducted in three independent tests, and data were presented as mean ± standard deviation (SD). For bioactivity analyses, multiple comparisons were carried out by one-way analysis of variance (ANOVA) and by Tukey's test with *p* = 0.05. Dissimilarities between means from at least three independent tests (*p* < 0.05) were judged statistically significant.

## Results

### Wine Pomace Stability

#### Comparison of Different Pomace Drying Methods

**Figure 1:**
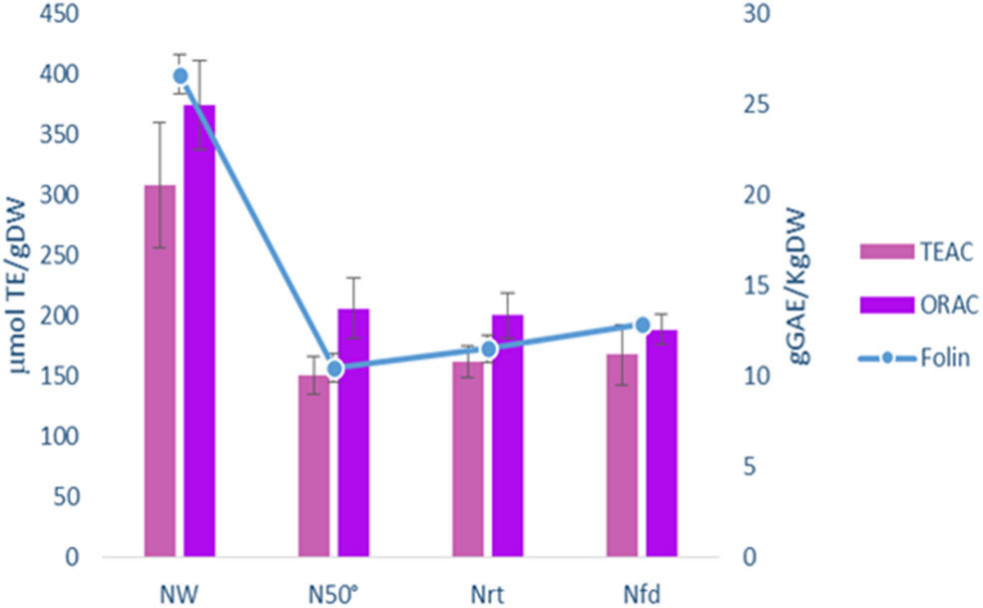
Comparison of Total Phenol (g GAEs/Kg DW Folin) and Antioxidant activity (μmoles/TE/g DW TEAC, ORAC) in grape skin from Pomace of Negramaro grape cv before (NW) and after dehydration with different methods: oven 50°C (N50°C); room temperature (N r.t.); freeze-dried (N f.d). Data are mean ± S.D. and are representative of three different assays performed.

In order to stabilize grape pomace (GP), we utilized different drying methods and then compared several parameters in the dehydrated matrix. Wet fresh pomace of Negramaro cv. was dried by oven at 50°C for 48 h (N50°), subjected to natural drying at room temperature for 72 h (N r.t.), or freeze-dried for 48 h (N f.d.). Subsequently, the skins were manually recovered from the grape pomace and stored at −20°C until further processing. As shown in Figure 1, the antioxidant activity and phenol content (Folin) of skin pomace dried with different methods (N50°, N r.t., N f.d.) showed similar results for all dehydration treatments. When compared to the wet material, the antioxidant activity of the skin pomace samples assayed by TEAC and ORAC methods was reduced by up to 50% in lyophilized and air-dried samples. The mean reduction in the total phenolic content was 49.3% for all drying treatments. Therefore, we selected the dehydration method by oven at 50°C because it is cheaper, faster, and more reliable than drying with air at room temperature (only 48 vs. 72 h).

## Characterization of Dried Skin Pomaces

### Extraction by Organic Solvents

In this study, we characterized the polyphenolic pattern and antioxidant properties of three different red GP, from Primitivo (P), Negramaro (N), and Negramaro/Lambrusco (N/L), and one white GP from Verdeca (B). Grape skin pomaces, separated from raw pomace dehydrated by oven at 50°C for 48 h, were analyzed for antioxidant activity (TEAC and ORAC) and total phenol content and compared.

**Figure 2:**
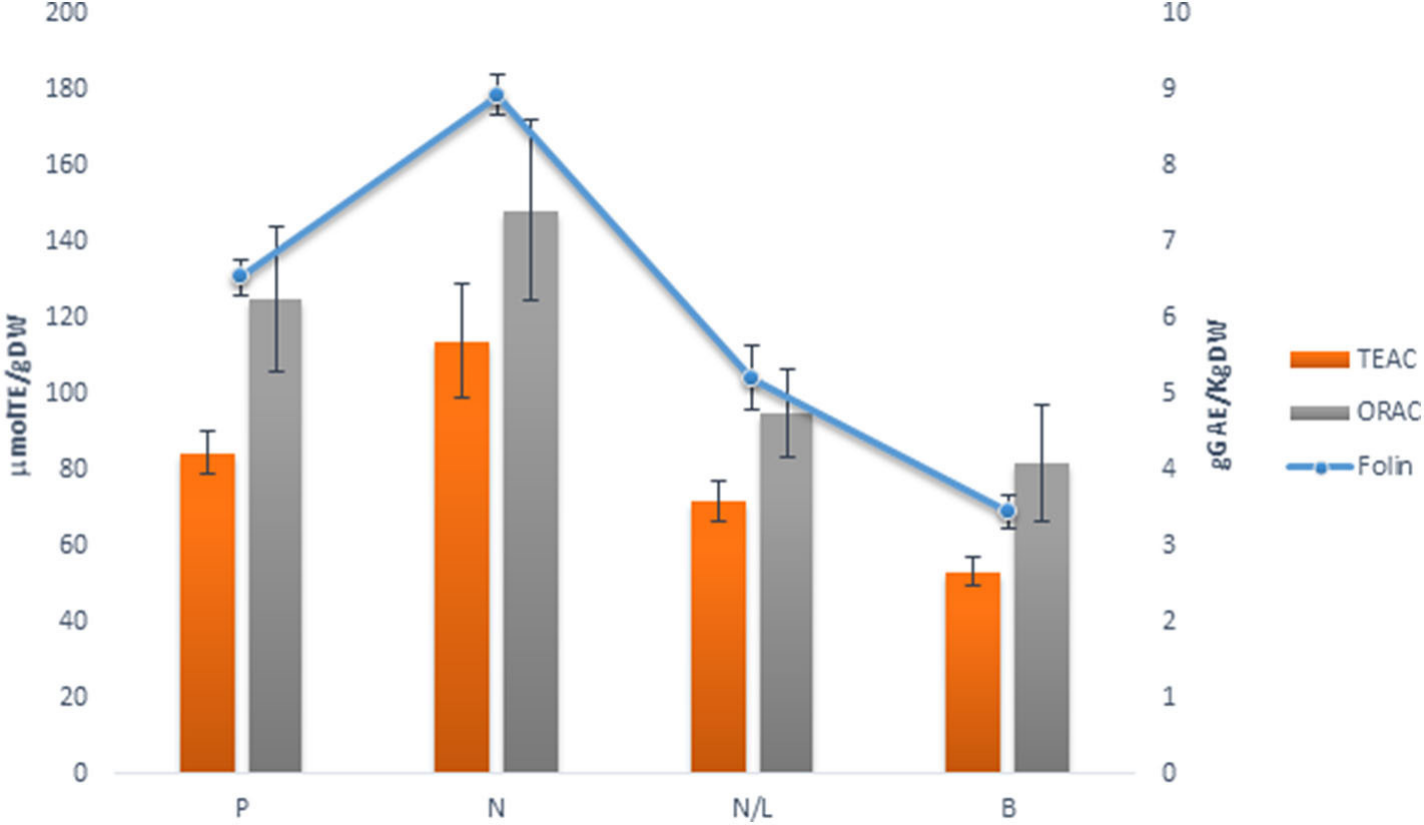
Comparison of Total Phenol (g GAEs/Kg DW, Folin) and Antioxidant activity (μmoles/TE/g DW, TEAC, ORAC) in different grape skin pomaces (Primitivo, P, Negramaro, N, Negramaro/Lambrusco, N/L, Verdeca, B) after dehydration at 50°C extracted in Methanol:ethanol 80:20, v/v. Data are mean ± S.D. and are representative of three different assays performed.

Both the total phenols (TP) and antioxidant activity (TEAC and ORAC) showed significant differences among GP skins (Figure 2). Indeed, N skin pomace showed higher values for both antioxidant activity (TEAC: 114.00 μmol TE/g DW; ORAC: 148.53 μmol TE/g DW) and TP content (8.94 g GAE/kg DW). TEAC, ORAC, and Folin-Ciocalteu assays indicated slightly lower values for P skin pomace, while the N/L blend showed the lowest values, similar to those for white GP skin (B), and for this reason was discarded from further analyses.

Polyphenolic composition was determined by solid-phase extraction in methanol/ethanol (8 : 2, v/v). Notably, anthocyanins were the most representative polyphenols in all the red extracts, followed by phenolic acids, flavonols, flavanols, and stilbenes (Table 1). Conversely, in white GP skin, a higher content of flavonols was recorded, followed by flavanols and phenolic acids (Table 1).

**Table 1:** Polyphenol contents (mg/Kg of dry weight) of grape skins recovered from winemaking in alcoholic extracts.

**Sample**	**Phenolic acids**	**Stilbenes**	**Flavanols**	**Flavonols**	**Anthocyanins**
**mg/Kg d.w**.					
P	568.89 ± 7.56	4.53 ± 0.01	236 ± 5.6	362.94 ± 13.5	6290 ± 0.25
N	954.40 ± 8.84	3.54 ± 0.33	200 ± 4.0	400.83 ± 4.84	7070 ± 0.17
N/L	781.07 ± 4.26	1.17 ± 0.26	180 ± 0.5	321.30 ± 7.02	2500 ± 0.20
B	104.28 ± 0.01	0	300 ± 2.2	407.69 ± 1.33	90 ± 0.01

Polyphenols (± SD) of grape skin from pomace of Primitivo cv (P), Negramaro cv (N), Negramaro/Lambrusco cv (N/L), and one white grape skin pomace of Verdeca cv (B).

*Results are average ± SD*.

Climatic conditions, viticulture practices, and the winemaking process can determine important variations in the mineral content of wine pomace more than for other components (Lachman et al., 2013). For example, the type and principally the duration of maceration processes have a strong impact on the extraction and reabsorption of minerals during winemaking. Table 2 reports the mineral content in different GP skins. Potassium (K) and calcium (Ca) were the most abundant micro-elements detected in all samples. More differences were found in white pomace (B) for magnesium (Mg) content, and in N samples for iron (Fe) content.

**Table 2:** Metal contents (mg/Kg of dry weight) of grape skin from pomace of Primitivo cv (P), Negramaro cv (N), Negramaro/Lambrusco cv (N/L), and one white grape skin pomace of Verdeca cv (B).

**Sample**	**Ca**	**Fe**	**K**	**Mg**	**Na**	**Zn**
**mg/Kg d.w**.						
P	3925.17 ± 149.80	246.25 ± 16.80	20587.25 ± 1094.20	395.81 ± 24.20	206.05 ± 10.50	14.18 ± 1.20
N	4258.06 ± 98.80	720.11 ± 17.40	17917.91 ± 108.20	506.14 ± 3.60	267.49 ± 0.20	10.99 ± 0.01
N/L	4062.52 ± 33.70	172.78 ± 0.70	24681.14 ± 704.30	443.12 ± 1.30	166.56 ± 4.80	9.48 ± 1.56
B	4067.87 ± 52.70	234.26 ± 7.20	19759.56 ± 48.70	831.44 ± 24.00	232.27 ± 3.20	8.1 ± 0.28

*std for blank sample: 0.003 (0 ± 0.003). Results are average ± SD*.

### Water Extraction

With the aim of utilizing the pomace skin powder to produce a functional drink, we set up a method to extract different polyphenol classes and other chemical compounds with functional activity using citric acid-acidified water. The three grape skin pomaces were treated for phytochemical recovery after water extraction. Two concentrations of skin powder (5 and 10%) were used to extract phytochemicals overnight (16 h) at 26°C. The results were similar for 5 and 10% grape skin powder (data not shown), suggesting a saturation effect during extraction. For this reason, further experiments were carried out using 5% grape skin concentration.

Preliminary results suggest that 5% pomace skin powder dissolved in water might be a good GP powder/water ratio. This water extract was used to characterize total anthocyanins, flavonols, and soluble acids among polyphenols together with organic acids, alcohol, and essential metals. To increase the polyphenol extraction rate in water, several different methods using ultrasound and/or microwave treatment were also used.

Antioxidant capability, TP, characterization of main polyphenol classes, organic acids, and metal analyses are reported in Tables 3–5. Table 3 shows that the aqueous extracts of B showed the highest content of flavonols but the lowest content of the remaining polyphenol classes, and lowest antioxidant activity and total polyphenol content. The N aqueous extract showed the highest antioxidant capacity, and total polyphenol and phenolic acid content, while the anthocyanin content of P was the highest for the pomaces extracted here in water. As reported in Table 4, tartaric acid was the most abundant organic acid in aqueous extracts of P, N, and B, followed by citric acid. The content of tartaric and citric acid was quite similar in the three analyzed extracts. Ethanol was not detectable in aqueous extracts. Characterization of the mineral contents in aqueous extracts of grape skins recovered from GP (Table 5) showed a high and similar content of both Ca and K and a higher content of Mg in B and of Na in P aqueous extract.

**Table 3:** Different Polyphenol group contents, Antioxidant Activity (AA) and Total Phenol (TP) of grape skins recovered from winemaking, in aqueous extracts obtained with acidified water.

**Sample**	**Polyphenols**				**AA**	**TP**
	**Phenolic acids**	**Stilbenes**	**Flavonols**	**Anthocyanins**		
**(w/v)**	**mg/L**				**mmolTE/L**	**mgGAEs/L**
P 5%	22.36 ± 0.301	0.19 ± 0.01	1.79 ± 0.37	14.21 ± 0.8	1.14 ± 0.03	107.80 ± 4.90
N 5%	43.63 ± 0.09	0.18 ± 0.05	1.66 ± 0.43	10.22 ± 0.9	2.00 ± 0.01	183.50 ± 11.90
B 5%	4.83 ± 0.20	0	3.77 ± 0.34	2.11 ± 0.5	0.87 ± 0.02	88.50 ± 0.29

Primitivo skin pomace (P), Negramaro skin pomace (N), and Verdeca white skin pomace (B).

*TE, Trolox Equivalents; GAE, Gallic Acid Equivalents. Results are average ± SD*.

**Table 4:** Organic acids and alcohols (g/L) from grape skins in aqueous extracts.

**Sample**	**Organic acids**				**Alcohol**	
	**Succinic acid**	**Malic acid**	**Tartaric acid**	**Citric acid**	**Glycerol**	**Ethanol**
**(w/v)**	**g/L**				**g/L**	
P 5%	0.10 ± 0.01	0.11 ± 0.01	2.17 ± 0.28	1.27 ± 0.01	0.65 ± 0.04	ND
N 5%	0.04 ± 0.00	0.25 ± 0.01	2.24 ± 0.20	1.54 ± 0.09	0.31 ± 0.14	ND
B 5%	0.19 ± 0.00	0.22 ± 0.01	1.95 ± 0.01	1.38 ± 0.03	0.42 ± 0.03	ND

Organic acids and alcohols (± standard deviation) of Primitivo skin pomace (P), Negramaro (N), and Verdeca white skin pomace (B).

*Detection limit for ethanol: 1.3 g/L. ND= Not detectable. Results are average ± SD*.

**Table 5:** Minerals (mg/L) in aqueous extracts of grape skins from pomace obtained with acidified water.

**Sample**	**Ca**	**Fe**	**K**	**Mg**	**Na**	**Zn**	**Cu**
**(w/v)**	**mg/L**						
P 5%	79.21 ± 3.80	0.25 ± 0.01	965.30 ± 0.30	25.17 ± 0.91	13.66 ± 0.10	0.54 ± 0.001	1.32 ± 0.01
N 5%	80.23 ± 1.23	0.33 ± 0.001	971.50 ± 23.30	25.28 ± 0.07	10.66 ± 0.01	0.22 ± 0.001	0.48 ± 0.001
B 5%	73.63 ± 0.17	0.39 ± 0.09	1139.45 ± 348.50	45.70 ± 12.13	6.82 ± 0.10	0.35 ± 0.14	0.29 ± 0.05
H_2_O	21.32	0.0041	4.502	1.621	3.102	0.0088	≤ d.l.

Minerals (± standard deviation) from 5% w/v, skin of Primitivo pomace (P), skin of Negramaro pomace (N), and skin of Verdeca white pomace (B).

*Cu detection limit: 0.001 mg/L. std for blank sample: 0.003 (0 ± 0.003). Results are average ± SD*.

### Comparison of Different Extraction Treatments in Water

We utilized traditional extraction methods generally based on a low heating process that, although enabling mass transfer among different phases of the extraction system, consume little energy, and prevent degradation of heat sensitive molecules.

To increase the polyphenol extraction yield and shorten extraction time, ultrasound and microwave methods have been investigated (Galanakis, 2013). These treatments aim to avoid/reduce the use of organic solvents, shorten the treatment time, decrease the temperature and energy consumption, increase the extraction yields, and preserve the final extract quality (Medina-Torres et al., 2017).

**Figure 3:**
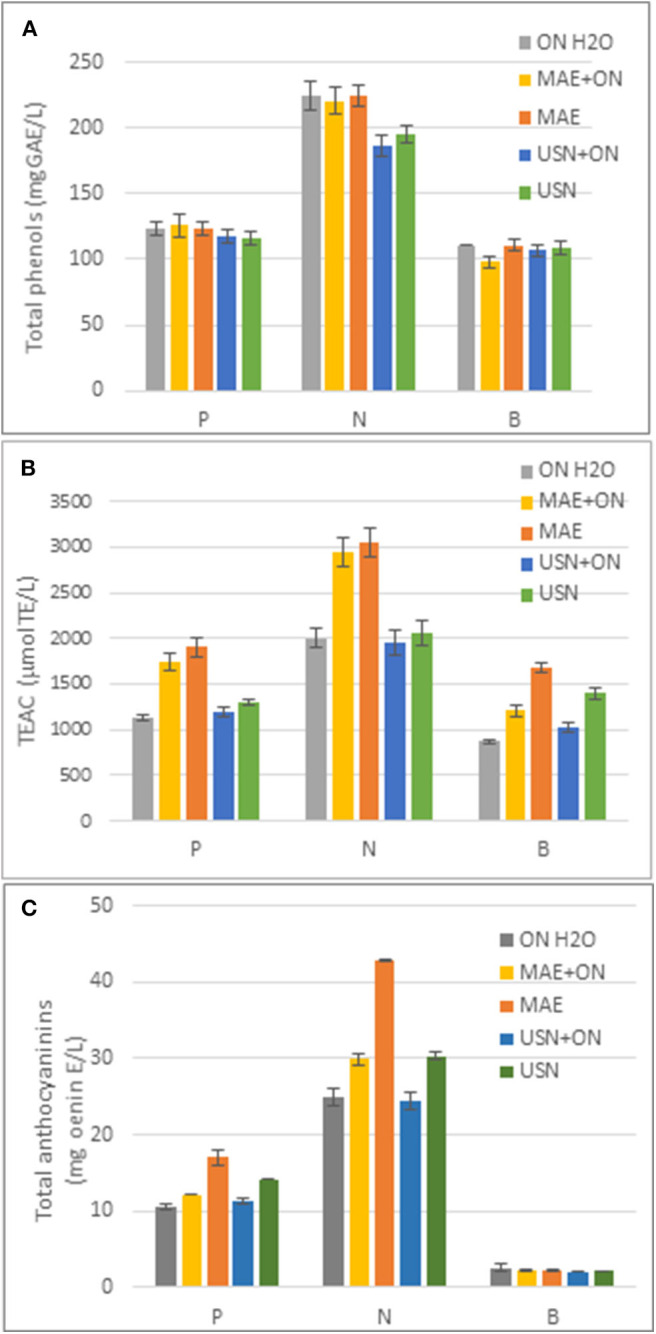
**(A)** Total Phenol (mg GAEs/L) in different grape skin pomace extracted in acidified water. P (Primitivo), N (Negramaro), B (Verdeca), at rt for 16 h on a rotatory shaker (ON H_2_O), with Microwave for 1 h at 50°C (MAE), MAE and 16 h, r.t. in a rotatory shaker (MAE+ON), with Ultrasound (UAE), UAE and 16 h, r.t. in a rotatory shaker (UAE+ON). **(B)** Antioxidant activity in different grape pomace skin (TEAC μmolesTE/L) extracted in water with methods described in **(A)**. **(C)** Total Anthocyanins in different grape pomace skin extracted in water with methods described in **(A)**. Data are mean ± S.D. and are representative of three different assays performed.

The TP content and antioxidant activity of extracts were determined to evaluate the effects of different extraction methods (Figure 3). Our results indicated a similar TP content (Folin in Figure 3) in samples subjected to UAE, MAE, and H_2_O extraction; these results confirmed that TP are extracted well in water, and UAE or MAE extraction treatments do not improve extraction in any case. An interesting result was obtained for the antioxidant activity recorded after MAE of all the skin pomace samples. Indeed, the antioxidant activity of MAE aqueous extracts was significantly higher (P: 68% more; N: 52% more; and B: 90% more). These data parallel with the amount of anthocyanins extracted, which was higher in the samples extracted by MAE in comparison to the controls extracted overnight at 26°C (P: 61% more and N: 71% more). It can be assumed that skin pomace samples with a higher anthocyanin content have a higher antioxidant activity.

## Production and Characterization of Infusions

### Antioxidant Activity, and Polyphenol, Organic Acid, and Essential Metal Content of Skin Pomace Infusions and Skin Pomace-Enriched Infusions

Despite the number of reports in the literature describing the anti-inflammatory, anticancer, and antioxidant activity of herb infusions, there are no clinical studies confirming their health-increasing properties. Probably, due to lower complexity, herbal infusions are a less suitable delivery vehicle compared to extracts of bioactive ingredients consumed in a controlled dosage (Yin et al., 2017). Therefore, the production of an infusion with a customary and reliable biological effect is a challenge (Tschiggerl and Bucar, 2012).

With the aim to reuse skin pomace powder as an ingredient for infusions and taking into account the results obtained using water extraction, skin pomace powder was incubated in hot water for 5 min. The infusion obtained was analyzed for TP content and related antioxidant properties. Table 6 shows the results of the analyses carried out on infusion of Primitivo skin pomace (P, 2 g) in 100 mL hot water, and infusion of blends of 1 g P and 1 g of tea (P/T), 1 g P and 1 g of rooibos (P/R), and 1 g of P and 1 g of hibiscus dried flowers (P/K) in 100 mL of hot water. The results indicated that the total polyphenol amount and antioxidant capability were significantly different among samples, with highest levels recorded for the blend P/T followed by P/R (Table 6). The ratios of antioxidant activity (AA) to TP content for P and the blends are 0.012 except for P/T for which the ratio is 0.016 (Table 6).

**Table 6:** Comparison of Antioxidant activity (AA) and Total Phenol (TP) in acidified aqueous infusions of grape skin pomace of Primitivo cv (P, 2% w/v), P/Tea (P/T, 1%+1%, w/v), P/Rooibos (P/R, 1%+1%, w/v), P/Karkadè (P/K, 1%+1%, w/v) blends.

	**P** **2%**	**P/T** **1%+1%**	**P/R** **1%+1%**	**P/K** **1%+1%**
AA (mmolTE/L)	2.56 ± 0.106	15.7 ± 0.502	5.6 ± 0.36	3.22 ± 0.27
TP (mgGAE/L)	200.8 ± 17.2	982.25 ± 70.7	469.5 ± 10.71	254.0 ± 22.0
AA/TP	0.012	0.016	0.012	0.012

*Results are average ± SD*.

Moving to mineral content, Table 7 reports the content in different GP skins infusion blends. Significant amounts of potassium were found in all infusions analyzed. Due to their relatively high mineral content, infusions obtained from wine pomace are interesting alternatives to increase the intake of minerals in the diet, especially potassium, which has essential functions in human health with particular beneficial effects on cardiovascular diseases.

**Table 7:** Minerals in acidified aqueous extracts of grape skins recovered from winemaking.

**Sample**	**Ca**	**Fe**	**K**	**Mg**	**Na**	**Zn**	**Cu**
**mg/L**							
P	80.74 ± 3.80	0.10 ± 0.01	512.80 ± 5.8	7.52 ± 0.38	7.72 ± 0.40	0.17 ± 0.001	0.68 ± 0.10
P/T	34.32 ± 0.10	0.04 ± 0.001	261 ± 2.0	1.97 ± 0.80	5.48 ± 0.30	0.20 ± 0.001	0.26 ± 0.01
P/R	74.72 ± 1.75	0.07 ± 0.001	30.3 ± 0.1	1.97 ± 0.80	2.26 ± 0.10	0.18 ± 0.001	0.46 ± 0.00
P/K	94.50 ± 2.30	0.25 ± 0.001	324.1 ± 3.3	3.65 ± 0.30	7.17 ± 0.35	0.36 ± 0.001	0.43 ± 0.09
H_2_O	21.32	0.0041	4.502	1.621	3.102	0.0088	≤ d.l.

Minerals (± standard deviation) in infusion of Primitivo grape skins pomace (P, 2% w/v), and P/Tea (P/T, 1%+1%, w/v), P/Rooibos (P/R, 1%+1%, w/v), P/Karkadè (P/K, 1%+1%, w/v) blends.

*Cu detection limit: 0.001 mg/L. std for blank sample: 0.003 (0 ± 0.003). Results are average ± SD*.

## Biological Activity of Infusions

### Anti-inflammatory Properties of GP Skin Infusions

Several studies have reported that red grape polyphenols possess desirable biological actions, including cardiovascular protection due to their anti-inflammatory effects and endothelial protection (Castaldo et al., 2019).

The vascular endothelium plays a crucial role in the formation and development of atherosclerotic plaque. Under the influence of inflammatory and atherosclerotic stimuli, the endothelium acquires new functional and phenotypic properties; it becomes adhesive against monocytes due to the overexpression of leukocyte adhesion molecules such as vascular cell adhesion molecule (VCAM)-1 and intercellular adhesion molecule (ICAM)-1.

With the aim to investigate the potential anti-inflammatory effect of aqueous extracts of red grape skin infusion of Primitivo pomace (P), we analyzed endothelial-monocyte adhesion, the first step essential in the development of atherosclerosis, as well as the stimulated expression of endothelial adhesion molecules in cultured human endothelial cells challenged with the pro-inflammatory cytokine TNF-α.

To evaluate the effects of P infusion on endothelial–monocyte adhesion, HMEC-1 cells were pre-exposed to aqueous extracts of grape skin infusion of Primitivo pomace (P) at several concentrations (1, 2.5, and 5 mg/mL) for 2 h before stimulation with TNF-α (10 ng/mL).

**Figure 4:**
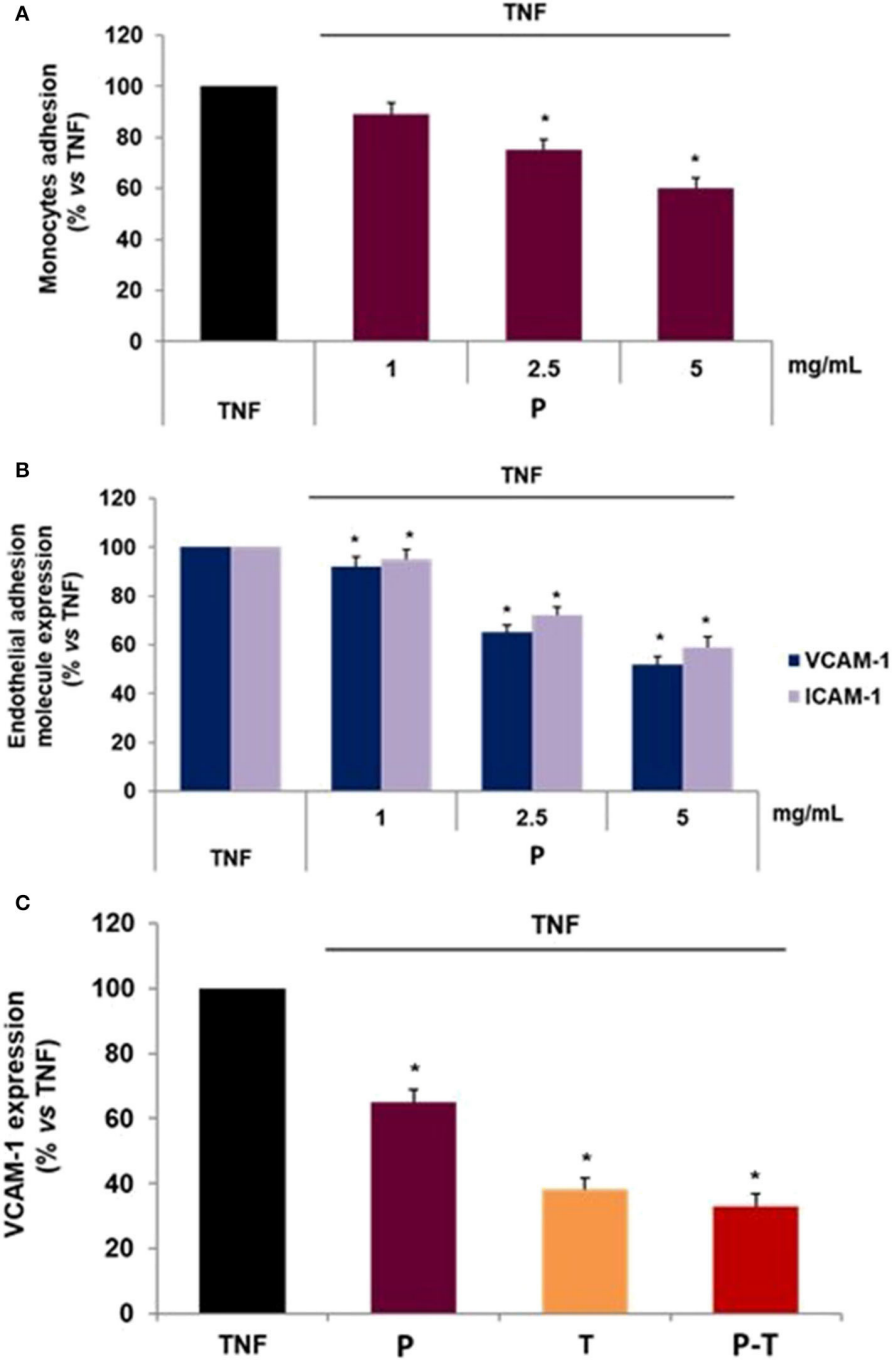
**(A)** Endothelial effect of grape pomace infusions. **(A)** Aqueous extracts of grape skins infusion of Primitivo pomace (P) decrease monocytoid cell adhesion to endothelial cells. U937 cells greatly adhere to endothelial cells challenged with TNF. P infusion decreases U937 cell adhesion in a concentration dependent manner. **(B)** Aqueous extracts of grape skin infusion of P inhibits the expression of TNF-stimulated expression of VCAM-1, and ICAM-1. **(C)** Aqueous extracts of grape skin infusion of blend P/Tea 1:1 (P/T) (2.5 mg/mL) reduces the surface expression of VCAM-1 in TNF-stimulated endothelial cells more than P or tea (T). Data are expressed as the percentage of TNF (mean ± S.D.) and are representative of three different experiments. *p < 0.05 vs. TNF alone.

Under an inflammatory challenge, mimicked by TNF-α, monocytoid U937 cells adhered strongly to TNF-stimulated endothelial cells (Figure 4). The treatment of HMEC-1 with P infusion, rich in polyphenols, significantly decreased TNF-induced monocyte adhesion in a concentration-dependent manner (Figure 4A), with an inhibitory effect evident from 2.5 mg/mL of infusion. These endothelial protective effects occurred in the absence of any sign of toxicity, as revealed by MTT and Trypan blue assays (data not shown).

Since the adhesion of monocytes to endothelium is related to the increased expression of endothelial adhesion molecules, we analyzed the effect of aqueous extracts of red grape Primitivo pomace infusion on the TNF-induced expression of VCAM-1 and ICAM-1, by cell-surface EIA (Figure 4B). P infusion reduced, in a concentration-dependent manner (1, 2.5, and 5 mg of freeze-dried infusion/mL), the TNF-stimulated expression of endothelial adhesion molecules ICAM-1 and even more of VCAM-1 (Figure 4B), with 1 mg/mL the lowest effective concentration.

We analyzed the anti-inflammatory properties of infusion obtained with grape skin Primitivo pomace (P) and tea (T) (1:1), named P/T. We compared the effect of the same concentration (2.5 mg/mL) of P/T with P and T on VCAM-1 expression in TNF-stimulated endothelial cells. We showed that the P/T blend is more effective in reducing endothelial VCAM-1 expression than P and T separately, lowering TNF-induced VCAM-1 expression by about 70% (Figure 4C).

This study has shown that the infusion of GP skin retains the endothelium-protective properties. The anti-inflammatory effect is even enhanced by the P/T infusion, suggesting its possible use as a functional beverage.

## Discussion

The valorisation of by-products is the priority to improve the sustainability of production chains through the reuse, recycling and recovery of energy. Moreover, the final use of a by-product should be integral, positively affecting human health or the environment (Galanakis, 2013). The direct use of wine chain by-products could be useful in the formulation of functional foods in order to improve consumer health. Mineral composition is also relevant to food production. For example, enzymes that are crucial in the quality of cereal products were stabilized by grape seed flours reach in calcium (Mironeasa et al., 2016). Furthermore, the exploitation of seasonings from wine pomace could improve the mineral content of foods, as it allows a reduction in the salt content. Products derived from grape and grape skin pomace provide interesting alternatives to increase the intake of minerals. For instance, sufficient potassium intake can contribute to control blood pressure and neuromuscular excitability (Rüdel et al., 1984; He and MacGregor, 2010).

To produce a sustainable beverage rich in GP skins, we have determined the conditions (time and temperature) that allow the extraction of the phenolic compounds using water as a solvent. The extracted bioactive molecules can find a wide range of applications in human nutrition, while the remaining organic residues in the extracts are not of concern.

Therefore, the drying process is important to stabilize and storage grape pomace. This drying step could enhance the potential use of pomace as a powerful natural antioxidant ingredient in functional foods. To avoid losing of bioactive compounds, due to their thermal instability, freeze drying is considered to retain higher levels of polyphenols than oven drying (Tseng and Zhao, 2012). As reported by Larrauri et al. (1997), no significant differences in total phenols were found either from freeze dryed or from oven dried (50°C) GP skin. Moreover, in our conditions, heat treatment over long-lasting periods may favorites the extractability of different low molecular weight compounds, increasing the level of phenolic compounds (Pedroza et al., 2012; Planinić, 2015). Drying by oven at 50°C was preferred because it allows the storage of grape skin from pomace and is faster and reproducible (Figure 1). The GP skins of two red grape varieties, Primitivo (P) and Negramaro (N), one blend of Negramaro/Lambrusco (N/L), and one white grape variety Verdeca (B) were stabilized at 50°C and their extraction in methanol/ethanol was characterized as reported in Figure 2 and Tables 1, 2. N and P extracts showed the highest AA and total polyphenol content, and an interesting composition of phenolic acids, stilbenes, and anthocyanins.

The metal content of GP skins is reported in Table 3 and shows that K is the most abundant metal in all varieties analyzed; similar findings have been reported by Pérez Cid et al. (2019) for grape skins isolated from the pomace of five Galician grape varieties.

The aim of the present work was to extract a significant amount of bioactive molecules, modifying the extraction solvent, finding a good compromise thus between quantity and quality. With the aim to utilize the pomace skin powder to produce a functional beverage, we set up a method to extract different polyphenol classes and other chemical compounds with functional activity in acidified water. Preliminary results suggest that 5% pomace powder dissolved in water is the right ratio of powder to water. As reported in Tables 3–5, we characterized total anthocyanins, flavonols and soluble acids among polyphenols, and four organic acids, alcohol, and metals in this water extract. As reported by Ferri et al. (2016) a lower amount of polyphenols are recovered from dried pomace in comparison to fresh pomace and organic solvents have a positive effect on their recovery.

Although it is necessary to improve the extractability of grape skin pomace in water, the content of these bioactive compounds and AA confirm grape skin pomace as a rich source of bioactive compounds with antioxidant and anti-inflammatory activity and suggest its exploitation as a functional ingredient.

In this study, grape skin extraction with acidified water was investigated in terms of maceration vs. UAE vs. MAE, to verify the differences and increase in the amount of polyphenols in water extract with the aim to produce a “ready-to-drink” functional beverage formulation. Actually, MAE and UAE extraction, even though considerably reducing the processing time, did not increase the amount of polyphenols in water, but the MAE process caused an increase in the AA and anthocyanin content in acidified water extracts. We conclude that these alternative techniques are interesting approaches that require study in more depth.

Several preclinical and clinical studies suggested that chemical synthesized or purified polyphenols do not provide the same biological activity as food matrix rich in the same compounds (Calabriso et al., 2016a,b; Scarano et al., 2018). With the aim to use grape skin isolated from GP to prepare infusions, in this study, hot water extracts were investigated. The AA, polyphenol content, mineral content, and anti-inflammatory effects of infusions formulated from Primitivo pomace skins alone or with added black tea, rooibos, or karkadè were evaluated (Tables 6, 7). As reported by Bekhit et al. (2011), the highest AA and polyphenol content were found in the infusion of grape skin pomace with added tea; moreover, the highest ratio of AA/TP points out that the P/T blend shows a higher AA of the TP fraction. This result is suggestive of a synergistic effect of the different classes of polyphenols contained in the blend. The polyphenols present in skin pomace water extract being rapidly absorbed, metabolized, and excreted, they exhibit low bioavailability. Nevertheless, accumulation of evidence that the beneficial activity of polyphenols occurs in humans is increasing. The potential synergism among different polyphenol metabolites could explain this argumentative result. For this reason, we have analyzed the health-promoting effects of infusions containing different types of bioactive molecules such as those present in grape skin pomace alone and with tea added. This study has shown that infusion of GP skin retain the endothelium-protective properties. The anti-inflammatory effect is even enhanced by the infusion of grape skin pomace and tea, suggesting a possible use as a functional beverage. Functional food/beverages development goal is to increase levels of health-promoting compounds human intake in order to deliver an efficacious amount. In a previous paper we demonstrated, by *in vitro* study, that polyphenols extracted from red grape skins exhibited beneficial effects at concentrations that are likely to be achieved in the plasma of subjects after moderate red grape skins consumption (Calabriso et al., 2016a). Moreover, the observed inhibitory effects of pure polyphenols, both flavonols and stilbenes, occurred at concentrations higher than those that can be found in red grape skin extracts, this suggests the occurrence of a synergism among different polyphenols in the extracts and that low doses of extracts could exhibit, also *in vivo*, synergic bioactive health-promoting effects. Moreover, factors that may improve polyphenols bioavailability is the glycosylation of the compound. Therefore, since grape skin pomace beverages contained stilbenes and flavonols mainly as glycosilated forms, it could be expected that they could be effective *in vivo* also at low doses.

## Conclusions

Studies on wine pomace demonstrate the potential exploitation of this by-product and suggest a new production chain for functional food production. Winemaking by-products can be reused as different food ingredients, such as fibers, polyphenol extracts, grape seed oil, and applied to produce new foods. However, investment costs for new food chain products are often high and a recovery strategy for the use of value products in functional food results in supplementary regulatory concerns. Thus, further scientific research is necessary to achieve significant advances in economic and regulatory problems. This work, substituting organic solvents with acidified water and using alternative techniques like UAE and MAE, meets the sustainable development concept since it reduces time and energy consumption. Our results clearly indicate the good quality of skin pomace acidified water extracts in term of polyphenol, organic acid, and mineral content. Moreover, infusions of grape skins alone and in combination with black tea were tested in cell models, showing anti-inflammatory activity. These attributes, together with its valuable AA, render grape skin pomace a noble by-product with great potential as an ingredient in health-promoting beverages and foods.

## Data Availability Statement

The datasets generated for this study are available on request to the corresponding author.

## Author Contributions

CG and GG conceived the work, analyzed data, and wrote and revised manuscript. GC performed HPLC, TEAC, ORAC, Drying methods. LD provided technical support. DM performed metals determination. ASal performed MAE extraction. MC determined biological activity and analyzed the data. ASan critically revised the manuscript. All authors approved the final version and made a substantial contribution to this work.

## Conflict of Interest

The authors declare that the research was conducted in the absence of any commercial or financial relationships that could be construed as a potential conflict of interest.
